# Pyroptosis executor gasdermin D plays a key role in scleroderma and bleomycin-induced skin fibrosis

**DOI:** 10.1038/s41420-022-00970-1

**Published:** 2022-04-08

**Authors:** Huan Yang, Yanqiang Shi, Huiting Liu, Feiyan Lin, Biying Qiu, Qinglan Feng, Yu Wang, Bin Yang

**Affiliations:** Dermatology Hospital, Southern Medical University, Guangzhou, 510091 China

**Keywords:** Immune cell death, Inflammasome

## Abstract

The NLRP3 inflammasome and IL-1β are essential for scleroderma pathogenesis. Nevertheless, the role of pyroptosis executor gasdermin D(GSDMD), which is a downstream molecule of NLRP3 and is required for IL-1β release in some situations, has not yet been well elucidated in scleroderma. Here, we found that GSDMD was significantly up-regulated and activated in the skin of scleroderma patients and bleomycin-induced mouse model. What’s more, the ablation of GSDMD ameliorates bleomycin-induced skin fibrosis according to HE staining, Masson staining and the detection of hydroxyproline contents. GSDMD deficiency also impaired macrophages infiltration and reduced inflammation response. Furthermore, the loss of GSDMD reduced Th17 differentiation in vivo and in vitro. Collectively, these findings provide the first demonstration that GSDMD related pyroptosis plays an important role in scleroderma pathogenesis.

## Introduction

Scleroderma is a broad term that denotes a series of diseases that manifest as skin fibrosis related to autoimmune dysfunction, including systemic sclerosis (SSc) and localized scleroderma (LoS) [1]. The pathological characteristics of skin in scleroderma are quite similar, which include microvascular damage, immune cells dysfunction, and fibrosis of the skin, except that internal organ fibrosis and systemic symptoms were involved in SSc [2, 3]. Both LoS and SSc show an early inflammatory and edematous reaction, which suggests that LoS and SSc may have similar inflammatory reactions at the initial stage [1, 2]. The most common symptoms of LoS are the sclerosis and atrophy of the skin, which can be disfiguring, and can have a certain risk of disabling when joints are involved. More importantly, SSc can affect the internal organs, which causes the high burden of mortality [4]. However, due to the complexity of this disease, effective therapies for scleroderma are still lacking. Therefore, it is very important to explore the pathogenesis of skin fibrosis in scleroderma and provide new insights for the treatment of the disease.

Studies have shown that inflammasomes play an important role in the pathogenesis of scleroderma [5]. Multiple inflammasome subsets have been reported to date, among which, NOD-like receptor family pyrin domain containing 3(NLRP3), is the most well studied. NLRP3 inflammasoms can recognize danger signals and activate the pro-inflammatory caspase-1 [6], which can cleave the precursors of IL-1β, IL-18 and Gasdermin D(GSDMD) into their mature form and induce pyroptosis, a programmed pro-inflammatory cell death. It is worth noting that in the skin lesions of SSc patients, the expressions of NLRP3 inflammasomes, caspase-1, IL-1β, and IL-18 were elevated, and they were positively correlated with the severity of skin fibrosis [7]. Furthermore, both NLRP3 knockout (KO) mice and ASC KO mice showed resistance to bleomycin (BLM)-induced skin fibrosis, highlighting an important role of NLRP3 in scleroderma [8].

Pyroptosis is a form of cell death mediated by the Gasdermin family, manifested by the destruction of cell membranes and the release of pro-inflammatory factors [9]. GSDMD is a classic pyroptosis executor in Gasdermins family. In the pyroptosis pathway mediated by GSDMD, inflammasomes can activate the pro-inflammatory caspase-1, which cleaves GSDMD and releases N-terminal-GSDMD. Then the assembling N-terminal-GSDMD transfers into cell membrane and forms membrane pores, promoting the release of cell contents and inflammatory mediators like IL-1β and IL-18. Broz P et al. [10] GSDMD has been confirmed to be related to many fibrotic diseases, such as non-alcoholic liver cirrhosis [11], renal fibrosis caused by ureteral obstruction [12] and myocardial fibrosis after myocardial infarction [13]. In addition, Peng L et al. [14] proved that Scutellarin can inhibit NF-κB/NLRP3 pathway to repress the activation of GSDMD and reduce BLM-induced lung fibrosis in mice. However, so far there have been no studies on the mechanism for GSDMD in BLM-induced skin fibrosis in mice and whether pyroptosis occurs in scleroderma patients.

Here, we provide the first insight into the physiological function of GSDMD in skin fibrosis of scleroderma and try to describe the regulatory mechanism of GSDMD-mediated pyroptosis in the development of immunological dysfunction in scleroderma patients and mouse model. We discover that GSDMD is crucial for skin fibrosis, and may be related to the infiltration of macrophages and differentiation of T helper 17 (Th17) cells in the skin lesions.

## Results

### Upregulation and activation of GSDMD in patients with scleroderma

**Fig. 1:**
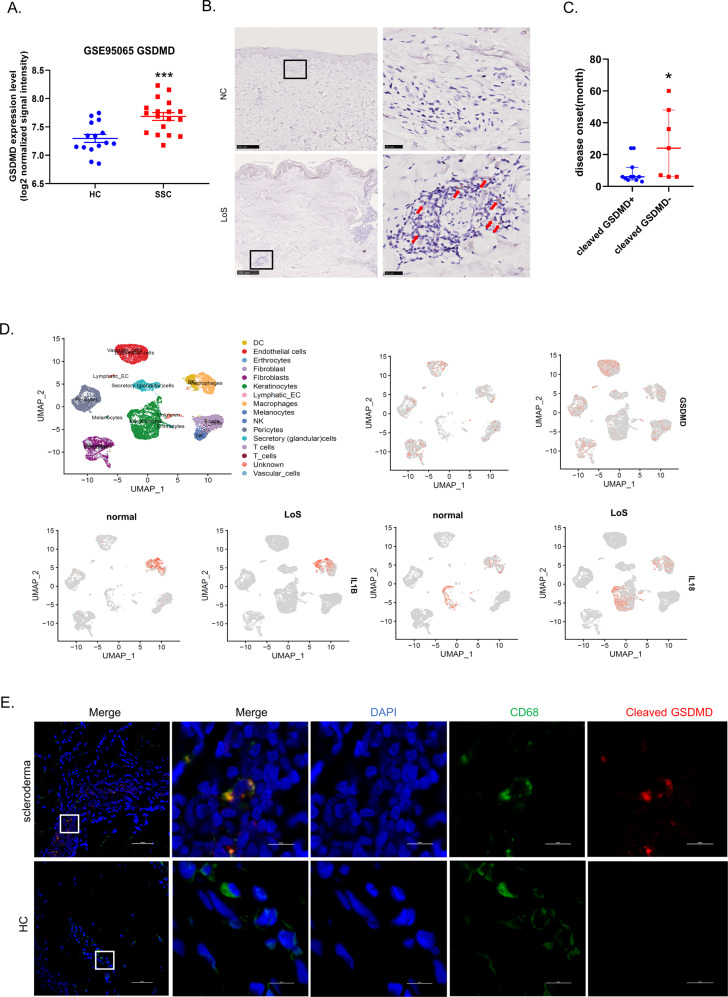
Upregulation and activation of GSDMD in patients with scleroderma. **A** Microarray data(GSE95065) of skin biopsies of patients with SSc (*n* = 18) demonstrated up-regulated mRNA GSDMD compared to healthy controls (HC) (*n* = 15), ***P < 0.001, analyzed by *T* test. **B** Representative images of three-micron thick skin tissue stained with anti-cleaved-GSDMD antibody, Red arrows: cleaved GSDMD positive cells. **C** Comparison of the disease duration of cleaved GSDMD positive and negative patients, the bars represent the median and IQR, *P < 0.05 (analyzed by Mann–Whitney *U* test). **D** Single cell RNA-seq data of skin biopsies of patients with localized scleroderma (*n* = 6) demonstrated the mRNA level of GSDMD, IL-1β and IL-18 were mainly up-regulated in macrophages, data also available at www.fibroad.org. **E** Immunofluorescence analysis using anti-cleaved-GSDMD and anti-CD68 antibodies for cleaved-GSDMD-positive macrophages. Scale bar: 50 μm and 10 μm.

To determine whether pyroptosis executor GSDMD plays a role in the development of scleroderma, we compared gene expression between healthy skin (*n* = 15) and SSc skin samples (*n* = 18) using microarray data in the Gene Expression Omnibus (GEO) database (GSE95065). The expression level of GSDMD was found to be significantly up-regulated in SSc skin than in normal skin (*p* = 0.0005) (Fig. 1A). In order to detect whether GSDMD induced pyroptosis occurs in scleroderma, we used immunohistochemistry staining to test the existence of the cleaved GSDMD in scleroderma skin with the excellent GSDMD antibody that recognize endogenous levels of GSDMD only when GSDMD is cleaved at Asp275. We enrolled a total of 19 patients, whose paraffin-embedded skin tissues were used for research, including 6 SSc and 13 LoS. The demographic and clinical manifestations of the included patients were shown in Supplementary Table S1. The results showed that there were cleaved GSDMD positive cells in some of the patients’ skin lesions, which were mainly distributed around blood vessels (Fig. 1B). However, not all patients have cleaved GSDMD positive cells in the skin lesions. Patients with longer disease duration and weaker inflammation have no obvious positive signals of cleaved GSDMD (Supplementary Table S1). It is also found that the disease duration of cleaved GSDMD-positive patients is generally shorter than that of cleaved GSDMD-negative patients (*P* < 0.05) (Fig. 1C, Supplementary Table S1). Patients with early-stage lesions which manifested as erythema or swelling were more likely to have cleaved GSDMD positive cells (Supplementary Table S1). Next, to explore what kind of cells have cleaved GSDMD in scleroderma skin, we downloaded and analyzed the GSE160536 data set, which included single-cell RNA sequencing (scRNA-seq) data from the skin lesions of six localized scleroderma patients. The analyzed data of GSE160536 are also available on www.fibroad.org. The results suggested that GSDMD (Fig. 1D), caspase-1 and caspase-4 (Supplementary Fig. S1) were mainly up-regulated in macrophages, vascular endothelial cells and T cells. What’s more, IL-1β, IL-18 (Fig. 1D) and NLRP3 (Supplementary Fig. S1) were mainly up-regulated in macrophages. Therefore, we further examined whether there was a GSDMD-related pyroptosis in macrophages in scleroderma. The result of immunofluorescence colocalization showed the presence of activated GSDMD in CD68-positive macrophages, suggesting that the pyroptosis of macrophages existed in scleroderma (Fig. 1E).

### Pyroptosis executor GSDMD is also up-regulated and activated in BLM-induced scleroderma mice

**Fig. 2:**
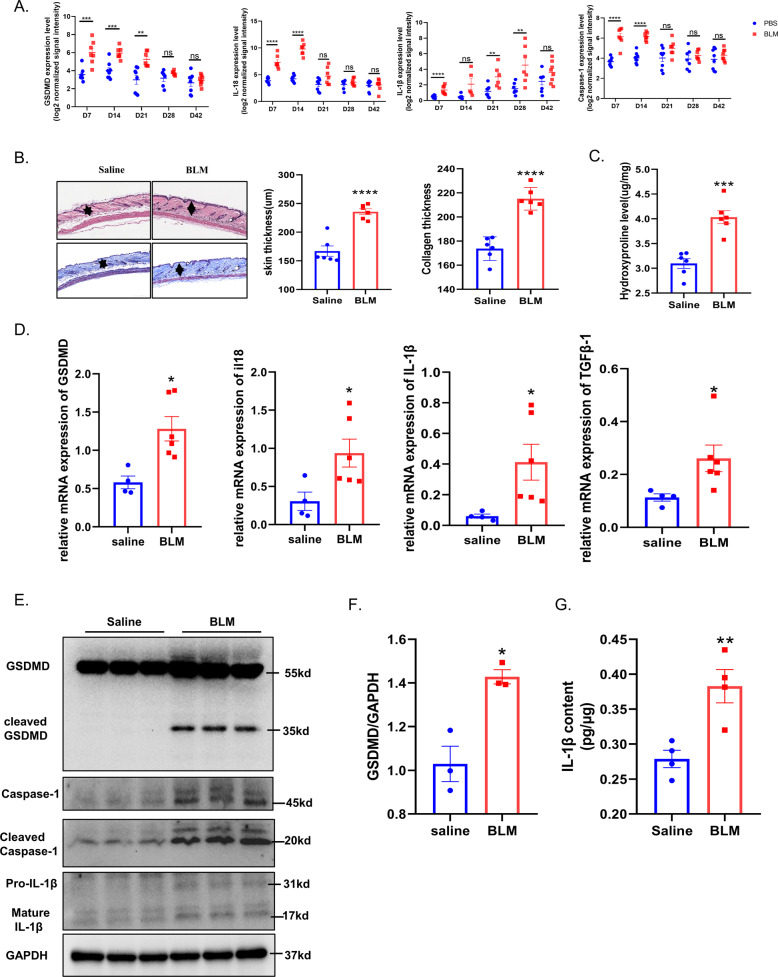
Pyroptosis executor GSDMD is also up-regulated in BLM-induced scleroderma mice. **A** RNA-seq data(GSE132869) of skin biopsies of mouse after treated with BLM at different time point demonstrated up-regulated GSDMD, IL-1β, IL-18, and Caspase-1 in the early stage. **B**, **C** Representative images of HE and Masson staining and Statistics on skin thickness, collagen thickness and hydroxyproline content showed that the BLM-induced skin fibrosis was successfully established (*n* = 6), the bars represent the mean ± SEM, ***P < 0.001, ****P < 0.0001 (analyzed by *t*-test). **D** Mouse skin treated with BLM (*n* = 6) or saline (*n* = 4) for 28 days were harvested and total RNA was isolated for RT-qPCR, GSDMD, IL-1β, IL-18, and TGFβ-1 expression was normalized against GAPDH expression, the bars represent the mean ± SEM. *P < 0.05 (analyzed by *t*-test). **E**, **F** Immunoblots showing the levels of GSDMD, cleaved GSDMD, Caspase-1, cleaved caspase-1, and IL-1β in the indicated groups. **G** ELISA showing the levels of IL-1β in tissue supernatant in the indicated groups, results are mean ± SEM, *n* = 4, **P < 0.01 (analyzed by *t*-test), compared to control.

The BLM-induced mouse skin fibrosis model has been widely used in the research of scleroderma. We first analyzed the RNA-sequencing (RNA-seq) results of mouse skin fibrosis induced by BLM from the GSE132869 data set, which included the RNA transcription of the mouse skin at day 7, 14, 21, 28, and 42 after BLM injection. We analyzed the expression level of GSDMD, IL-1β, IL-18 and caspase-1 in this data set. Interestingly, we found that the expression of GSDMD, caspase-1, IL-1β, and IL-18 increased significantly at the early stage (days 7 and 14) of the disease in scleroderma mice. However, after 21 days, the expression of these genes gradually decreased into the level of the control group (Fig. 2A). This was similar to the findings of immunohistochemistry of cleaved GSDMD in scleroderma patients, suggesting GSDMD may mainly play a role at the initiation and early inflammatory stage of the disease. In order to further verify the role of GSDMD-induced pyroptosis in mice with scleroderma, we used BLM to establish scleroderma mouse model for study. BLM was injected subcutaneously at a fixed position on the back of the mouse, once every other day, for 4 consecutive weeks. The results of HE staining, Masson staining, skin thickness measurement and hydroxyproline contents showed the successful establishment of the mouse model (Fig. 2B, C). The skin samples were then used for RNA and protein extraction. The results of qPCR showed that after BLM treatment, the transcription levels of GSDMD, IL-1β, IL-18 and, the classic molecules of fibrosis, TGFβ-1, were increased (Fig. 2D). The results of Western Blot showed that the full-length GSDMD and pro-IL-1β was up-regulated in protein level (Fig. 2E, F). Moreover, the protein levels of cleaved-GSDMD, cleaved caspase-1 and mature IL-1β were also elevated in scleroderma mice (Fig. 2E, F). ELISA test of the tissue supernatant found that the level of IL-1β in the skin of the BLM treated group was increased, which further proved the existence of GSDMD-related pyroptosis in scleroderma mice (Fig. 2G).

### The ablation of GSDMD ameliorates BLM-induced skin fibrosis

**Fig. 3:**
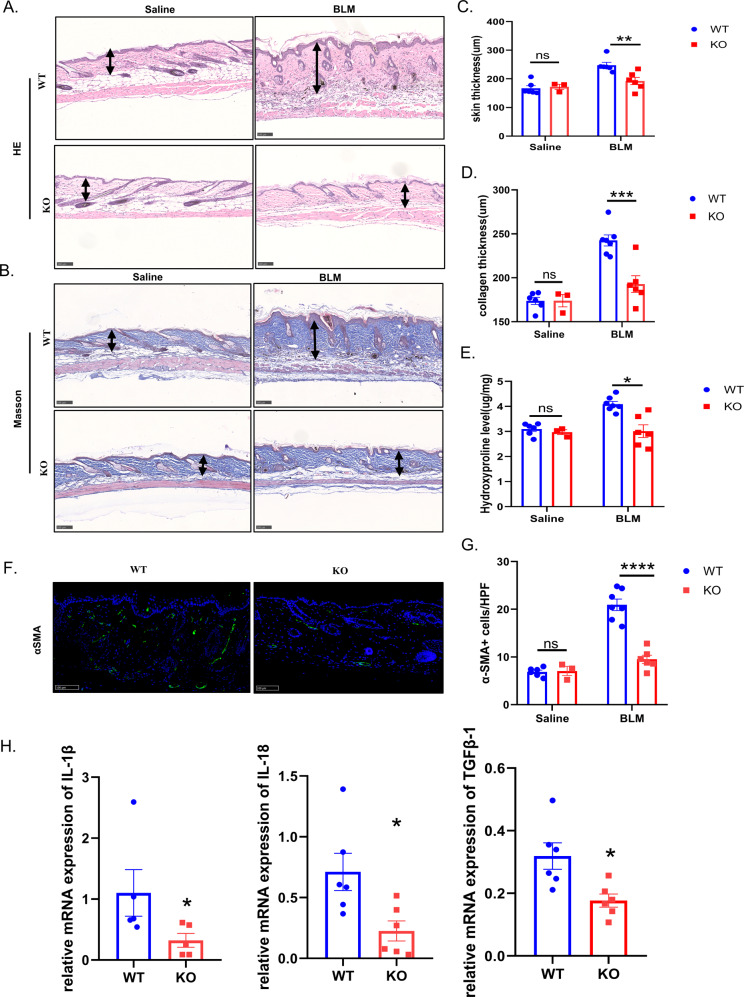
The ablation of GSDMD ameliorates BLM-induced skin fibrosis. GSDMD KO and WT mice received subcutaneous injections of saline or BLM every other day for 4 weeks, and lesional skin was collected and analyzed. **A**, **B** Representative images with HE and Masson staining of GSDMD WT and KO treated with Saline (*n* = 6, *n* = 3) or BLM (*n* = 7, *n* = 6). Black arrows represent the distance measurement. Scale bar: 100 μm. **C**–**E** Quantitation of dermal thickness, collagen thickness, and hydroxyproline content demonstrating decreased dermal thickness in GSDMD KO mice injected with BLM compared with WT mice, results are mean ± SEM, *P < 0.05, **p < 0.01, ***P < 0.001, ****P < 0.0001 (analyzed by *t*-test). **F**, **G** myofibroblasts staining by anti-α-SMA antibody in per high power field, results are mean ± SEM, ****P < 0.001 (analyzed by *t*-test). **H** RT-qPCR analysis in the skin lesions from WT and GSDMD KO mice treated with BLM for 4 weeks, results were normalized against GAPDH, the bar were mean ± SEM, *P < 0.05 (analyzed by *t*-test).

To further investigate the role of GSDMD in skin fibrosis, gender- and age-matched GSDMD KO mice and WT control mice were subcutaneously injected with BLM, and then indicators related to skin fibrosis were analyzed. The results of HE and Masson staining showed that GSDMD KO mice had an attenuated skin fibrosis and proliferation of collagen fibrous tissue, and immune cells infiltration was significantly decreased compared with WT mice (Fig. 3A, B). The thickness of the skin and collagen was significantly less in GSDMD KO mice (*P* < 0.0001) (Fig. 3C, D). The hydroxyproline content in the skin tissues of GSDMD KO mice was significantly lower than that in WT mice (*P* < 0.05) (Fig. 3E). The number of α-SMA-positive myofibroblasts significantly reduced in GSDMD KO mice compared with WT mice (*P* < 0.0001) (Fig. 3F, G). The transcription levels of IL-1β, IL-18, and TGFβ-1 also decreased (Fig. 3H). Disulfiram can inhibit the cell membrane poring activity of GSDMD [15]. It reminds us that disulfiram may be able to reduce the degree of fibrosis in mouse skin, so we injected disulfiram into the intraperitoneal cavity 4 hours before BLM administration. The results of HE staining and Masson staining showed that the skin thickness of mice in the disulfiram administration group was significantly less than that in the control group. (Supplementary Fig. S2A). The results of hydroxyproline and α-SMA detection also showed that the skin collagen content of mice in the disulfiram administration group was less (Supplementary Fig. S2B–D). This suggested that disulfiram might have a therapeutic effect on skin fibrosis.

### Knockout of GSDMD attenuated immuno-response and reduced macrophages infiltration

**Fig. 4:**
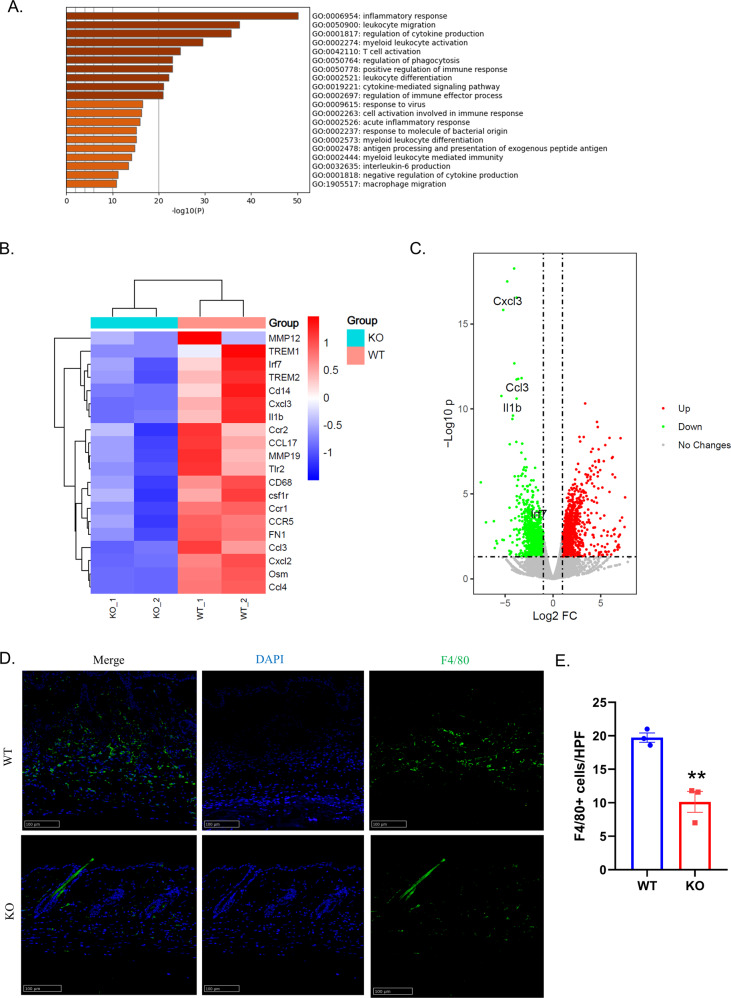
Knockout of GSDMD attenuated immuno-response and reduced macrophages infiltration. **A** GO analysis of RNA-seq data of skin biopsies of GSDMD KO and WT mouse skin after treated with BLM, *n* = 2. **B**, **C** RNA-seq data demonstrated CXCLs, CCLs, CCRs, MMPs, CD68, CD14, CSF1r, IL-1β, FN1, and IRF7 were down regulated. **D** Immunofluorescence analysis using anti-F4/80 for macrophages in BLM-treated mice skin, Scale bar: 100 μm. **E** Statistical analysis of the number of macrophages per high power field, results are mean ± SEM, (*n* = 3), **P < 0.01 (analyzed by *t*-test).

To elucidate the mechanism for how the GSDMD influent the fibrosis process, we performed RNA sequencing (RNA-seq) and compared the mRNA expression profiles of total RNA extracted from GSDMD KO mice and WT mice after BLM administration for 28 days. Gene Ontology (GO) analysis revealed that most of the differentially expressed genes were associated with inflammatory response, macrophage migration and activation, as well as T cell activation (Fig. 4A). The heat map showed that many chemokines were significantly down-regulated in GSDMD KO mice compared with WT mice, including CXCL3, CXCL2, CCL2, CCL3, CCL4, CCL17, and chemokine receptors CCR1, CCR2, CCR5 (Fig. 4B, C). In addition, the expressions of monocyte or macrophage surface markers like CD14, CD68 and csf1r were down-regulated, suggesting a decrease in the number of macrophages in GSDMD KO mice (Fig. 4B). Immunofluorescence showed that the number of macrophages in the skin lesions of GSDMD KO mice was significantly reduced (*P* < 0.01) (Fig. 4D, E). The results of RNA-seq also showed that the expressions of cytokines IL-1β and OSM and the key transcription factor IRF7 [16] in scleroderma were down-regulated in GSDMD KO mice (Fig. 4B, C).

### Decreased Th17 response in GSDMD KO mice in BLM-induced scleroderma murine model

**Fig. 5:**
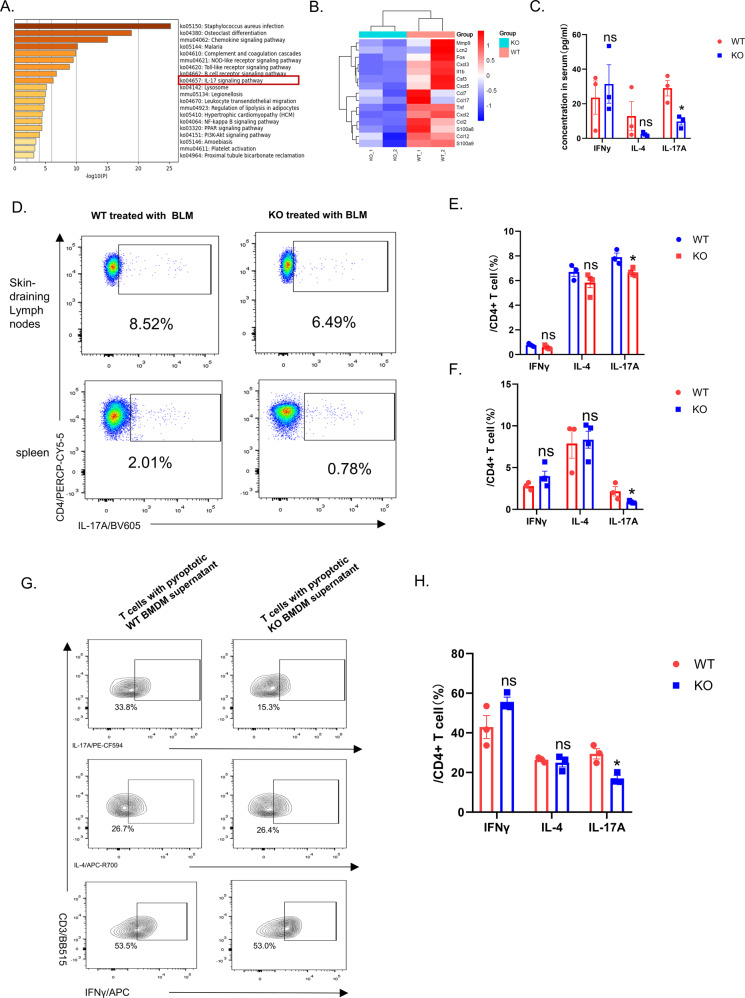
Decreased Th17 response in GSDMD KO mice in the BLM induced animal model. **A** KEGG pathway ananlysis of RNA-seq data of skin biopsies of GSDMD KO and WT mouse skin after BLM challenge, *n* = 2. **B** Heat map showing the genes with altered expression that were related to the IL-17 pathway. **C** The content of IFN-γ, IL-4 and IL-17A in peripheral blood serum of GSDMD KO mice and WT mice treated with BLM for 28 days, results are mean ± SEM, *n* = 3, *P < 0.05 (analyzed by *t*-test). **D**–**F** Percentage and statistical analysis of CD4^+^ T cells that secret IFN-γ, IL-4, IL-17A in spleen and lymph nodes in GSDMD KO (*n* = 4) and WT (*n* = 3) mice after BLM-treated for 28 days, results are mean ± SEM, *P < 0.05 (analyzed by *t*-test). **G** CD4^+^ T were used for cytokine stimulation and flow cytometry after co-cultured with the supernatant of pyroptotic BMDMs. **H** Statistical analysis of cytokine secretion of the T cells in the indicated group; results were mean ± SEM, (*n* = 3), *P < 0.05 (analyzed by *t*-test).

We next performed Kyoto Encyclopedia of Genes and Genomes (KEGG) pathway analysis and identified multiple downregulated signaling pathways including IL-17 signaling pathway (Fig. 5A). Heat map showed the down-regulated genes related to IL-17 pathway (15 genes) in GSDMD KO mice (Fig. 5B). The detection of multiple cytokines in peripheral blood serum of mice indicated that the expression of IL-17A was significantly down-regulated in GSDMD KO mice (*P* < 0.05) (Fig. 5C). These suggest that GSDMD may promote the process of fibrosis by changing the immune response of Th17. We then further tested the cytokine secretion function of T cells in the skin draining lymph nodes and spleen of GSDMD KO and WT mice after subcutaneous BLM injections for 28 days. The results showed that the proportion of IL17A secreted by CD4^+^ T cells in GSDMD KO mice in the spleen and lymph nodes was significantly reduced compared with WT (*P* < 0.05) (Fig. 5D–F). We next extracted GSDMD KO and WT mouse BMDMs and used LPS and nigericin to induce pyroptosis. CD4^+^ T cells derived from the spleen of normal WT mice were then cultured with the supernatant of pyroptotic BMDMs and function of cytokine secretion of the T cells was tested. The results showed that the secretion of IL-17A from T cells that co-cultured with GSDMD KO BMDM treated with LPS and nigericin decreased (*P* < 0.05), but IFN-γ and IL-4 did not change significantly (Fig. 5G, H). This also proved our conjecture that GSDMD-mediated pyroptosis promotes the secretion of IL-17A by T cells, and further promote the occurrence of fibrosis.

## Discussion

There have been some preliminary studies on GSDMD in BLM-induced pulmonary fibrosis and skin fibrosis. Ling Peng et al. [14] demonstrated that BLM could up-regulate the levels of cleaved GSDMD, IL-1β, and IL-18 in lung fibrosis murine model. Liang Q et al. [17] found that lycorine could inhibit NLRP3, repressed the pyroptosis induced by GSDMD and alleviated the pulmonary fibrosis in the BLM-induced idiopathic pulmonary fibrosis (IPF) murine model. However, the physiological role and mechanism of GSDMD in the pathogenesis of BLM-induced skin fibrosis in mice and whether pyroptosis occurs in scleroderma patients are still unclear. Our study carefully explored the important role of pyroptosis executor GSDMD in scleroderma patients and BLM-induced skin fibrosis in mice for the first time.

In this study, we first demonstrated that the levels of GSDMD were up-regulated and cleaved GSDMD was present in the skin of scleroderma patients. Interestingly, we observed that cleaved GSDMD-positive cells were mainly scattered near the perivascular area of scleroderma skin lesions, some of them were macrophages, which proved that GSDMD induced pyroptosis occurs in scleroderma patients. Next, we found that GSDMD is also up-regulated and activated in BLM-induced scleroderma mice model. What’s more, with BLM challenge, GSDMD KO mice have reduced skin fibrosis compared with WT mice.

To explore how GSDMD affects skin fibrosis induced by BLM, we performed RNA-seq of the mice skin of GSDMD KO and WT mice. The GO analysis of our RNA-seq revealed that the major down-regulated immune pathways in BLM-treated GSDMD KO mice were associated with macrophage migration, T cell activation, antigen processing and presentation, and cytokine signaling (Fig. 4A), which indicated that the activity of GSDMD was involved in the activation of innate and adaptive immunity in scleroderma. The heatmap summarized that the loss of GSDMD impaired the expression level of chemokines and its receptors including CXCL3, CXCL2, CCL2, CCL3, CCL4, CCL17 and CCR1, CCR2, CCR5, which can control the migratory patterns of immune cells such as the recruitment of leukocytes to infected or damaged tissue [18], regulate immune activation of T cells [19], and play an important role in autoimmune diseases [20]. CCL2 and CCL3 have previously been found to be elevated in SSc [21]. CCL2 and CCL3 take participant in T cell activation and CCL2 promotes T cell differentiation into Th17 during inflammation [19, 22]. It is also suggested that CXCL3, CCL4, CCL17, CCR1, CCR2, CCR5 play great important roles in SSc through regulating immune response [23–27]. The results of RNA-seq also showed that the cell surface markers of monocytes and macrophages, like CD68, CD14 and Csf1r were down regulated in GSDMD KO mice. The further immunofluorescence results confirmed the decrease of macrophages in the skin of GSDMD KO mice. Monocytes and macrophages are important regulatory cells of the innate immunity, and are also an important participant in autoimmune diseases [28]. Moreover, macrophages can participate in tissue fibrosis by secreting a variety of cytokines, including TNF-α and IL-1β and undertaking macrophage-to-myofibroblast Transition [29, 30]. Therefore, the reduction of macrophages in GSDMD KO mice may contribute to the repression of inflammatory response and tissue fibrosis. In general, GSDMD KO reduces the expression of chemokines and their receptors, inhibits the infiltration of macrophages, suppresses the immune inflammatory response of the tissue, and attenuates fibrosis.

GSDMD is required for IL-1β secretion in BLM-induced skin fibrosis and pharmacological inhibition of the GSDMD attenuated fibrosis. The release of IL-1β is important for GSDMD-related pytoptosis to participate in innate immunity. What’s more, IL-1β plays an important role in the pathogenesis of SSc [31], and it can synergize with TGF-β2 and drive endothelial to mesenchymal transition(EMT) [32]. From our results of RNA-seq and RT-qPCR, Western Blot and ELISA, we can make reasonable assumptions that BLM activates GSDMD and promotes the upregulation and secretion of IL-1β, while genetic ablation of GSDMD reduces the expression of IL-1β in BLM-induced scleroderma mice. Disulfiram is a FDA-approved drug that used clinically to treat alcohol addiction. It has been reported that disulfiram can specifically inhibit the cell membrane poring activity of GSDMD, thereby inhibiting pyroptosis and IL-1β release [15]. In this study, we also found that disulfiram can inhibit BLM-induced fibrosis. Although disulfiram is an unspecific inhibitor with a wide range of target molecules, it may still be a potential treatment for skin fibrosis.

It has also been reported that level of IL-17 in SSc patients was significantly increased [33], and IL-17A could mediate the inflammatory response of endothelial cells in SSc by regulating the ERK pathway, and promote the progression of fibrosis [34]. In addition, IL-17+IFNγ + Th17 cells were significantly increased in SSc patients, and could promote the progression of fibrosis by secreting IL-21 [35]. In BLM-induced skin and lung fibrosis models, IL-17A and Th17 cells could promote the proliferation and cytokines secretion of fibroblasts, thereby promoting the process of fibrosis [36]. KEGG pathway analysis of our RNA-seq results found that some of the differentially expressed genes were enriched in the IL-17 pathway. We cultured CD4+ T cells with the supernatant of pyroptotic BMDMs in vitro, and the resulting T cells had a stronger ability to secrete IL-17A, indicating that GSDMD-related pyroptosis in macrophages could promote T cells differentiate into Th17, which may contribute to skin fibrosis in scleroderma.

This study is the first to study the role of GSDMD in the pathogenesis of skin fibrosis in scleroderma. However, the findings of the present study have to be seen in light of some limitations: (1) This study focused more on the analysis of the RNA levels of GSDMD-related molecules, and the protein levels of IL-1β, IL-18, and some other molecules in the skin lesions of GSDMD KO and WT mice are still needed. (2) Pivotal experiments are still lacking to demonstrate the important role of IL-1β and IL-17 in skin fibrosis. Neutralization of IL-1β and adoptive transfer of Th17 cells in GSDMD KO mice are needed. What’s more, low concentrations and short time points of nigericin treatment group are required to make the experiment more rigorous. (3) The mechanism by which disulfiram affects skin fibrosis is not clear, and still needs further detailed research.

In conclusion, our study provides insights into physiological role of GSDMD as a key mediator in the pathogenesis of scleroderma and suggests GSDMD and its related molecules as potential therapeutic targets.

## Materials and methods

### Patients

We searched for patients diagnosed with scleroderma, whose clinical records were retrievable, from a computerized pathology database at the Dermatology Hospital of Southern Medical University between 2018 and 2020. We selected 19 cases, 6 SSc, 10 plaque-type LoS, and 3 linear-type LoS (Supplementary Table S1). Paraffin-embedded tissue blocks of these patients were retained for study. The paraffin blocks of Healthy controls (HC) were borrowed from the sample library of Dermatology Hospital of Southern Medical University. The demographics and characteristics of HC were demonstrated in Supplementary Table S2.

### Mice

GSDMD KO mice were purchased from cyagen company (Guangzhou, China). C57BL/6 fertilized egg cells were co-injected with Cas9 mRNA and gRNA in vitro to create GSDMD KO mice. The mice were housed in specific pathogen-free facility with 12 h light/dark cycle. The animal experiments were conducted according to the protocol approved by the Institutional Animal Care and Use Committee of Southern Medical University (Guangzhou, China).

### Murine model of scleroderma

Age- and gender-matched WT C57BL/6 and GSDMD KO mice were used for the experiment. Skin fibrosis of scleroderma was established in 6 to 8-week-old female C57/6 J mice using BLM subcutaneous injection. Mice were anesthetized and 100 microliters of 2 U/ml BLM was subcutaneously injected to a fixed position on the mouse back every other day, for a total of 28 days. Subcutaneously injection of saline in mice was used as controls. Mice of the same genotype were randomly assigned to saline group or BLM group.

### Histologic analysis of skin sections

Skin samples were fixed with 4% paraformaldehyde for 24 h before they were embedded in paraffin. Paraffin sections (3 μm) were stained with hematoxylin-eosin (HE) and Masson’s Trichrome for morphology and fibrosis assessment. The images of HE and Masson staining were scanned and analyzed by NanoZoomer S60(Hamamatsu, Japan).

### Immunohistochemical and immunofluorescence staining

Paraffin sections were dewaxed and rehydrated according to standard protocol, and antigen retrieval was conducted by microwave in 1X antigen retrieval solution (Gene Tech) followed by antigen blocking. Human sections were then incubated with rabbit anti-human cleaved GSDMD (Asp275) monoclonal antibody (1:100; cell signaling technology, #36425), mouse anti-human CD68 monoclonal antibody (1:200; abcam, #ab955) at 4 °C overnight. Mouse sections were incubated with mouse anti-mouse α-SMA (1:30000; abcam, #ab7817) or rabbit anti-mouse F4/80(1:2000; proteintech, #28463-1-AP) at 4 °C overnight. Goat anti-mouse IgG-Alexa Fluor® 488 (1:700, abcam, #ab150113) and donkey anti-rabbit-Alexa Fluor® 555(1:700, abcam, ab150074) were used as secondary antibodies. Cell nucleus was labeled with DAPI (abcam, #ab104139). The specimens were then ready for visualization and images were acquired using a confocal microscope (Nikon A1+, Tokyo, Japan). For immunohistochemical staining, HRP-linked anti mouse or rabbit IgG antibody were used as secondary antibody, and 3,3N-Diaminobenzidine Tertrahydrochloride (DAB) was used for color developing. The images of immunohistochemical staining were scanned from NanoZoomer S60(Hamamatsu, Japan).

### Hydroxyproline analysis

Total collagen content of skin tissue samples was quantified using a hydroxyproline assay kit (#A030-2-1, Nanjing Jiancheng Bioengineering Institute, China) according to the manufacturer’s instructions. Briefly, Alkaline hydrolysate was added to skin tissue, followed by hydrolysis at 95 °C for 20 min. Then we adjusted the pH value of the samples to 6.0–6.8, and used activated carbon to remove impurities in the samples. The samples were then incubated with chloramine-T solution for 10 min at room temperature. Each sample was treated with perchloric acid solution for 5 min and then Dimethylaminobenzaldehyde (DMAB) was added to the reaction system 15 min at 60 °C for color development. Samples were read in a 96-well plate at 550 nm by a multiple microplate reader (Varioskan LUX, Thermo Scientific, USA).

### Western blot analysis

In order to detect the cleaved GSDMD and Caspase-1 in BLM-treated mouse skin tissues, the whole skin tissue was harvested and lysed with RIPA lysis buffer containing protease inhibitor and phosphatase inhibitor and 5xSDS was added to the lysate. The lysates were then put at 95 °C for 10 min, separated on 10% SDS-PAGE and transferred to polyvinylidene fluoride membranes. The membranes were blocked for 1 h in 5% milk at room temperature and incubated overnight at 4 °C with the rabbit anti-mouse GSDMD (1:1000; abcam, #ab219800), rabbit anti-mouse Caspase-1(1:1000, cell signaling technology, #24232), rabbit anti-mouse cleaved Caspase-1(1:1000, cell signaling technology, #89332), rabbit anti-mouse IL-1β (1:1000, cell signaling technology, #31202). After washing, the membranes were incubated for 1 h with horseradish peroxidase labeled secondary-antibodies at room temperature. The blots were visualized with ECL.

### Cell culture

First, to obtain mouse CD4+ T cell, we prepared single cell suspension using the spleen of WT mice, and then used naïve CD4+ T cell isolation kit (miltenyi biotec, #130-104-453) to sort out CD4+ T cells. RPMI1640 supplemented with 10% FBS, 2 ug/ml anti-CD28 antibody, 100 U/ml penicillin, and 100 mg/ml streptomycin (Invitrogen, #15140-122) was used to culture the sorted cell for 3–4 days in CD3 coated 96-well plate. To obtain mouse bone marrow derived macrophages (BMDMs), bone marrow cells derived from femurs, tibias and humerus were harvested and cultured with RPMI-1640 supplemented with 10%FBS, 50 ng/ml M-CSF (Peprotech, Rocky Hill, NJ), 100 mg/ml streptomycin, and 100 U/ml penicillin for 6 days. BMDMs were treated with 100 ng/ml LPS for 4 h, then remove supernatant and added culture medium with10 μM nigericin for 2 h to induce pyroptosis. CD4 + T cells were cultured with the supernatant of the pyroptotic BMDMs for 48 h, and then were harvested for flow cytometry.

### Flow cytometry

Total single cells were isolated from spleen and lymph nodes and were stimulated with leukocyte activation cocktail (1:500 in RPMI-1640 containing 10% FBS, BD bioscience, #550583) at 37 °C in a humidified incubator with 5% CO2. After 4 h of stimulation, cells were washed and incubated with TruStain FcX™ (anti-mouse CD16/32) antibody for Fc receptors blocking (BioLegend, #101320) and then exposed at 4 °C to a mixture of the following antibodies: Fixable viability stain 780 (1:1000, BD bioscience, #565388), anti-mouse CD4 (1:40, BD bioscience, #566407), anti-mouse CD3 (1:100, Biolegend, Clone 145-2C11). For cytokine secretion staining, after cell surface marker and viability staining, the cells were fixed and permeabilized. And then, the cells were incubated with anti-mouse IL17A (1:40, BD bioscience, #564169), anti-mouse IL-4 (1:40, BD bioscience, #564006), anti-mouse IFNγ(1:40, BD bioscience, #554413). Cells were analyzed with BD FACS Celesta (BD Bioscience). Flow cytometry analysis was performed using FlowJo software (TreeStar, Ashland, OR). The gating strategy is shown in Supplementary Fig. S3.

### ELISA

Twenty-eight days after BLM administration, the mouse skin samples were taken, grinded and resuspended in PBS, and then centrifuged to prepare tissue supernatant. Protein concentration in tissue supernatant was quantified by BCA protein assay. IL-1β level was quantified using the mouse IL-1 beta Uncoated ELISA Kit (Invitrogen, #88-7013) according to the manufacturer’s protocol. The final IL-1β level was adjusted to protein concentration in tissue supernatant.

### Real time-quantitative PCR

Total RNA was isolated from mouse lesional skin using RNAiso reagent (TAKARA), and then reverse transcribed into cDNA using the PrimeScript RT reagent kit with gDNA eraser (Takara Biomedical, #RR047A). Quantitative PCR was performed on a LightCycler 384 real-time PCR system (Biorad, CFX384Touch). Primers for target genes are shown in Supplementary Table S3. The TB Green premix Ex Taq II was used according to the recommended guidelines (TaKaRa, #RR820, Tokyo). The melting curves and values were assessed with the Bio-RAD software. Target gene expression levels were quantified by 2-ΔΔCT method using human GAPDH as the internal standard. Each sample was analyzed with three technical replicates.

### RNA-seq analysis

Total RNA was extracted from samples of GSDMD KO and WT mouse skin after treated with BLM, using TRIzol (15596; Invitrogen, USA). For transcriptomic analysis, the RNA quality of each sample was assured by 2200 Bioanalyzer (Agilent Technologies, Palo Alto, CA, USA) and NanoDrop (Thermo Fisher Scientific Inc.) before moving on to cDNA synthesis and library construction (Novaseq instrument according to manufacturer’s instructions (Illumina, San Diego, CA, USA)). A total of 106.3 million raw paired-end reads was obtained, averaging 26.5 million per sample. Reads were aligned to the reference genome mm9 with Hisat2 (v2.0.1). SAM files were generated from alignment results using SAM tools. Read counts were obtained with Featurecount (v2.0.1) with the union option. Differential expression was performed with the R/Bioconducter package DESeq2 by applying an adjusted *P* < 0.05 and an absolute log2 ratio larger than 1. Raw data and analyzed data are also available at National Center for Biotechnology Information (NCBI Gene Expression Omnibus GSE196637).

### Expression data analysis

Regarding RNA-seq data from C57BL/6 mice with/without BLM treatment, the expression file with count values was downloaded from GSE132869. Then raw counts of all samples were FPKM normalized. The scatter plot of genes expression analysis was generated by using GraphPad Prism v8 with the log2 FPKM. For single-cell RNA-sequence(scRNA-seq) data analysis, we downloaded the scRNA-seq data from GSE160536, which included six morphea patients. This data was analyzed using the Scanpy package with the reference genome GRCh38. Then, unified popular approximation and projection algorithm was used to reduce the dimension of the data, and then the Louvain algorithm were used to perform cluster analysis on the data. The gene expression was calculated according to the absolute value of the logarithm of gene expression ratio, so as to achieve the purpose of analyzing the expression of the target gene.

### Statistical analysis

According to the distribution of residuals, the experimental groups are compared by *t* test or Mann–Whitney *U* test. For the validity of the experimental statistics, each experimental group had no less than three samples. Data are presented as mean ± SE of the mean (SEM) when *t* test was used. When Mann–Whitney *U* test was used, the results are presented as dot plot with the median and interquartile range(IQR). Difference were considered to be statistically significant when two-tailed *p* values ≤ 0.05. Statistical analysis was and mapping were performed by Prism v8.0 software (GraphPad, CA, USA).

## Supplementary information

original wetern blot

supplementary materials

## Data Availability

The datasets generated and analyzed during the current study are available from the corresponding author on reasonable request.
