# Supplementary material for: Pyroptosis executor gasdermin D plays a key role in scleroderma and bleomycin-induced skin fibrosis
Source: Cell Death Discov. 2022 Apr 8;8:183. doi: 10.1038/s41420-022-00970-1 (PMC8993883; doi:10.1038/s41420-022-00970-1)

Original western blot

GAPDH

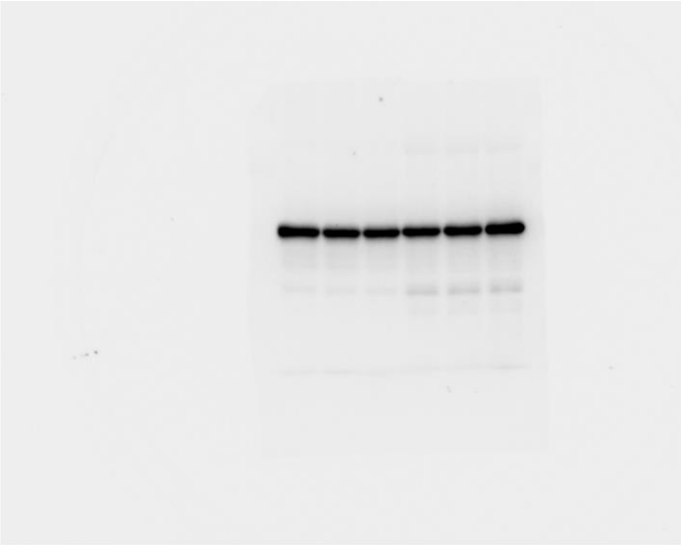

Gasdermin D

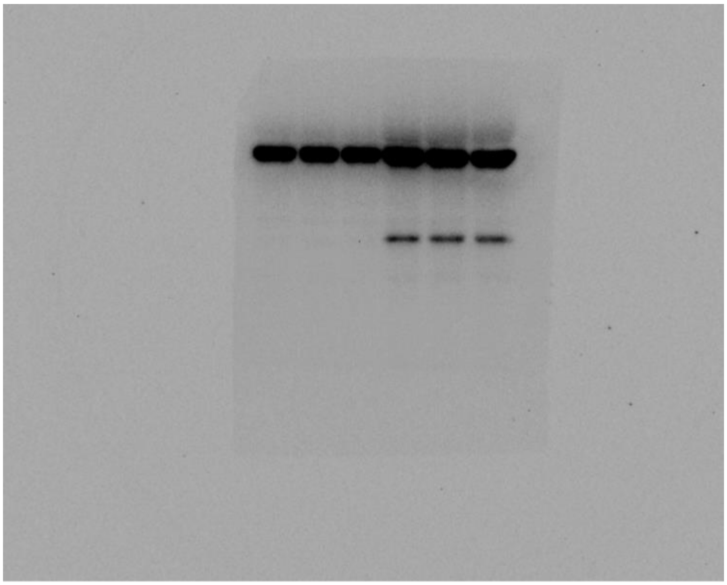

Caspase-1

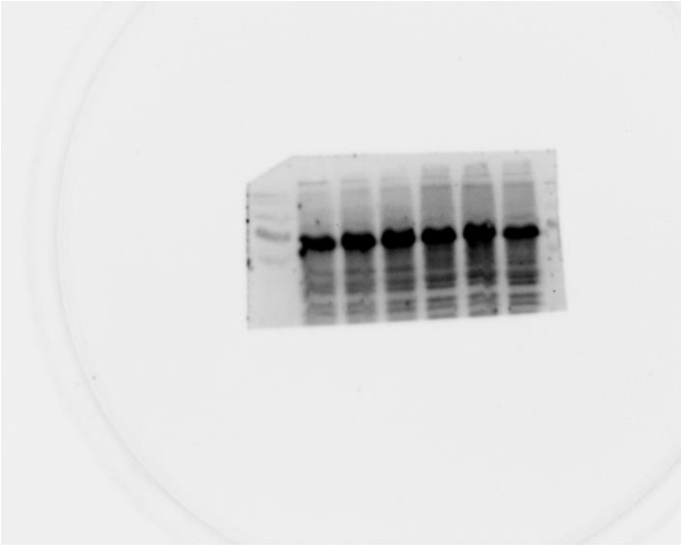

Caspase-1 merged with marker

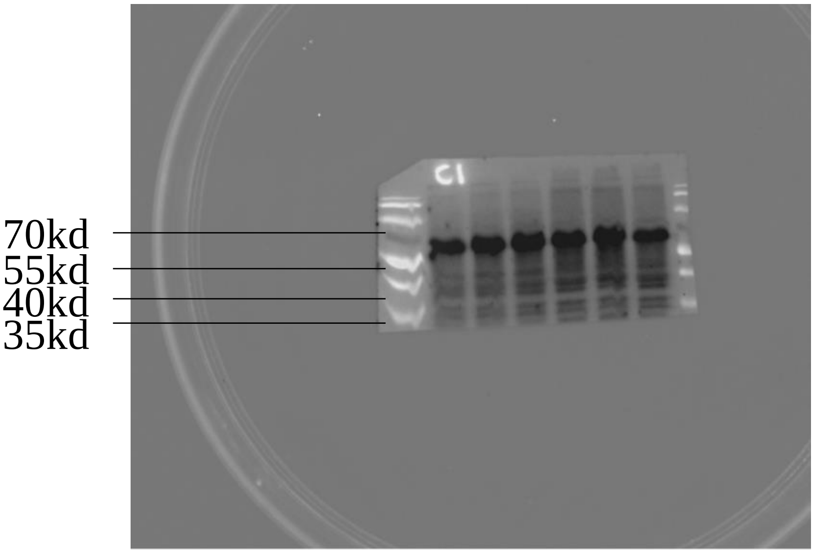

Cleaved Caspase-1

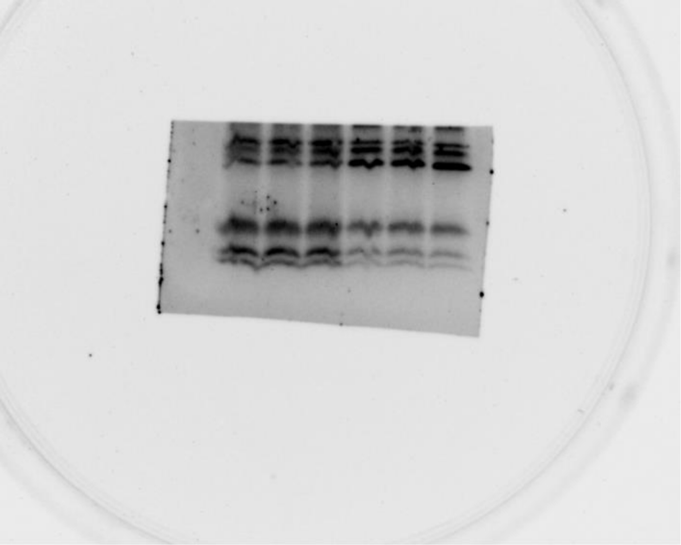

Cleaved Caspase-1 merged with marker

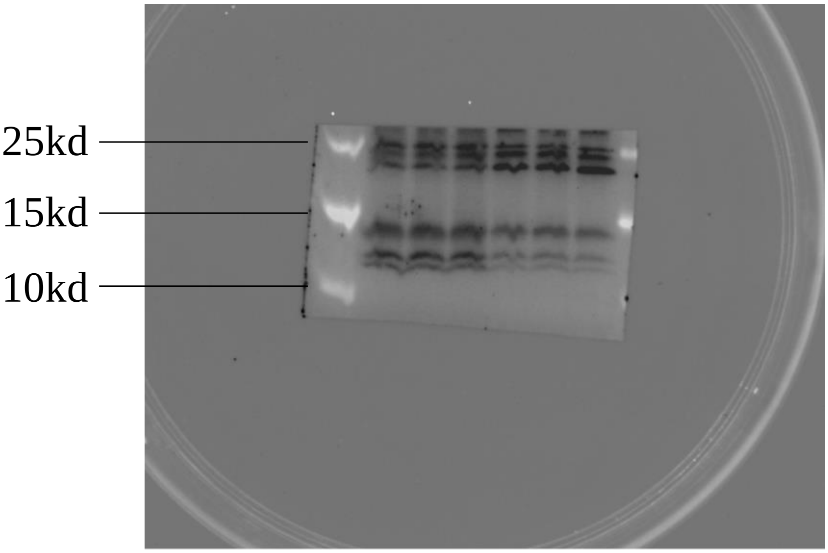

IL-1b

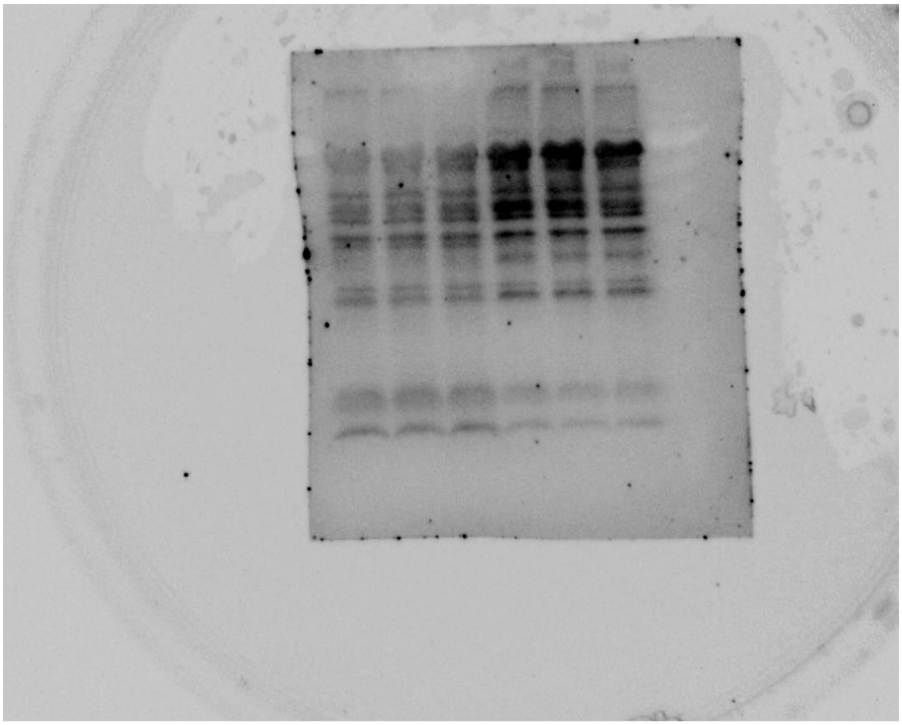

IL-1b merged with marker

35kd
25kd

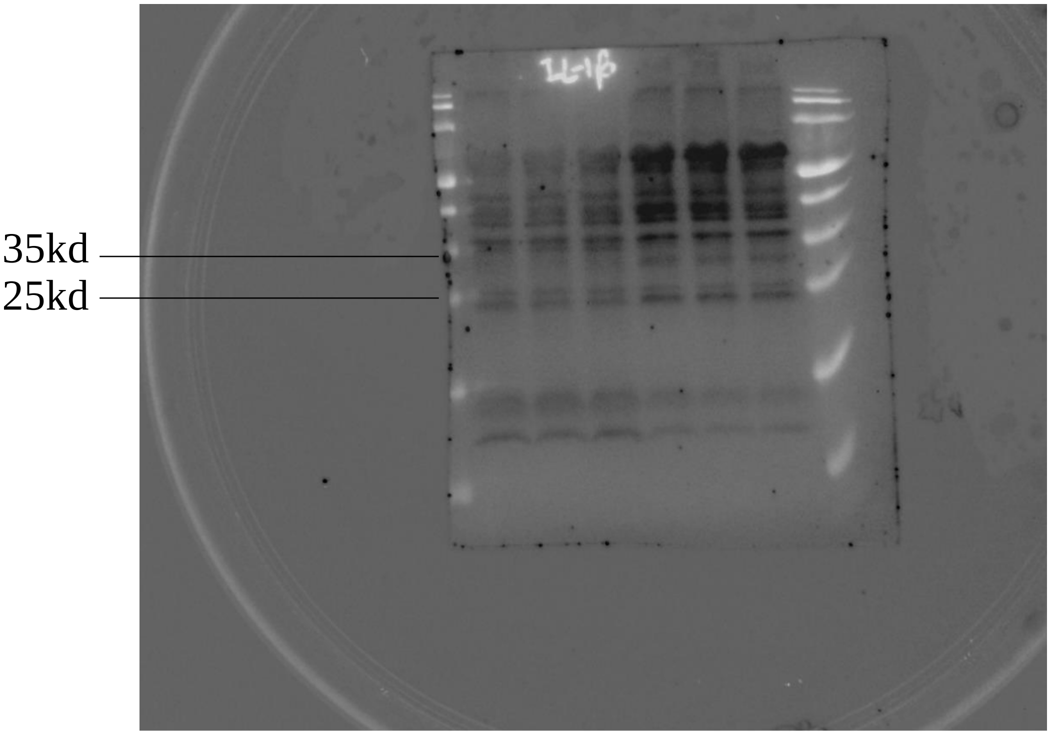

Supplement: Supplementary file 1 — original wetern blot [file 41420_2022_970_MOESM1_ESM.pdf]
