# Supplementary material for: Pyroptosis executor gasdermin D plays a key role in scleroderma and bleomycin-induced skin fibrosis
Source: Cell Death Discov. 2022 Apr 8;8:183. doi: 10.1038/s41420-022-00970-1 (PMC8993883; doi:10.1038/s41420-022-00970-1)
Supplement: Supplementary file 2 — supplementary materials [file 41420_2022_970_MOESM2_ESM.pdf]

Figure S1. Single cell RNA-seq analysis of Caspase-1, Caspase-4 and NLRP3 in localized scleroderma patients.

A.

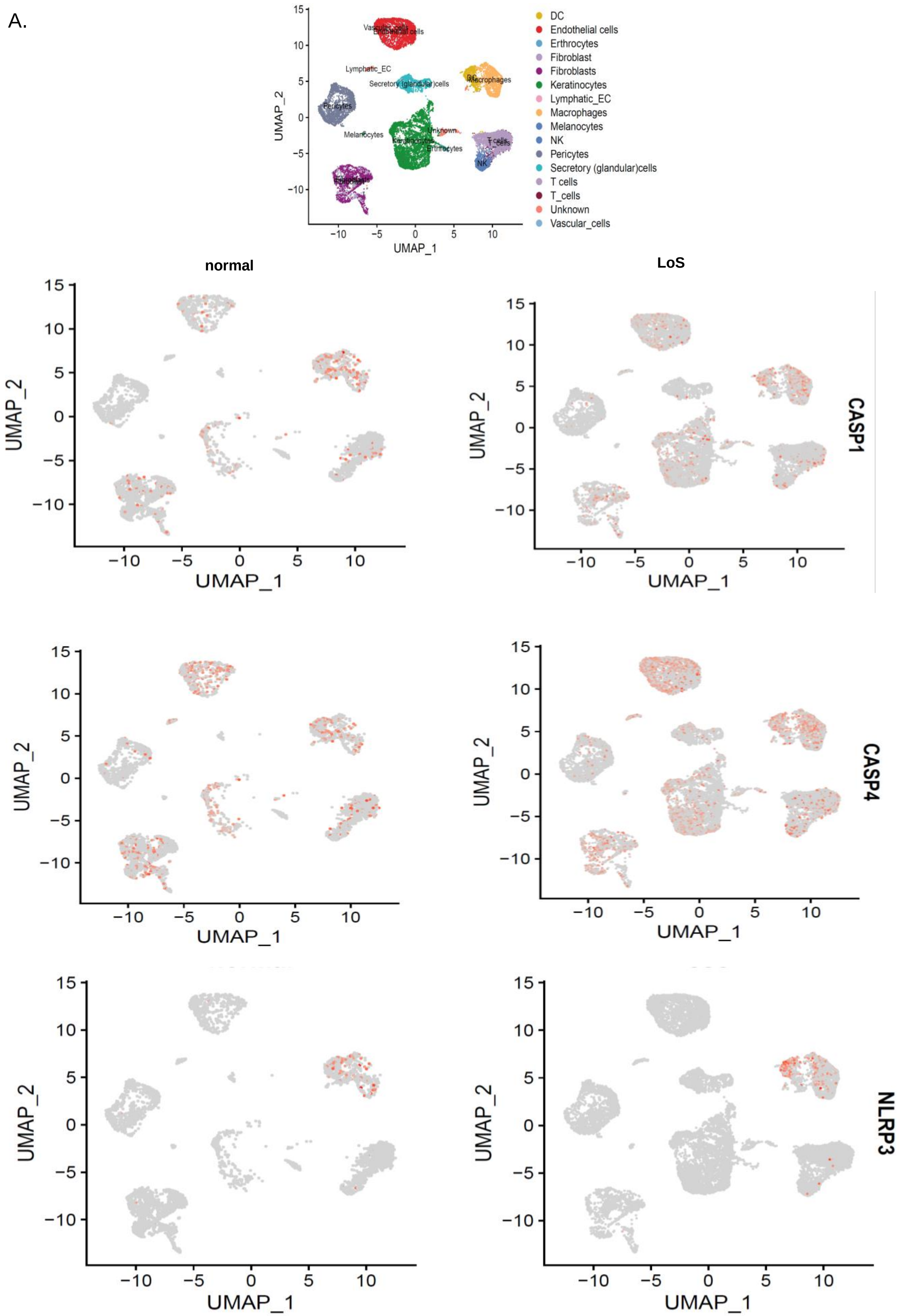

(A) Single cell RNA-seq data of skin biopsies of patients with localized scleroderma(n=6) demonstrated mRNA Caspase-1, Caspase-4 and NLRP3 mainly up-regulated in vascular endothelial cells, macrophages and T cells, data also available on www.fibroad.org.

Figure S2. Disulfiram administration reduces skin fibrosis in mice.

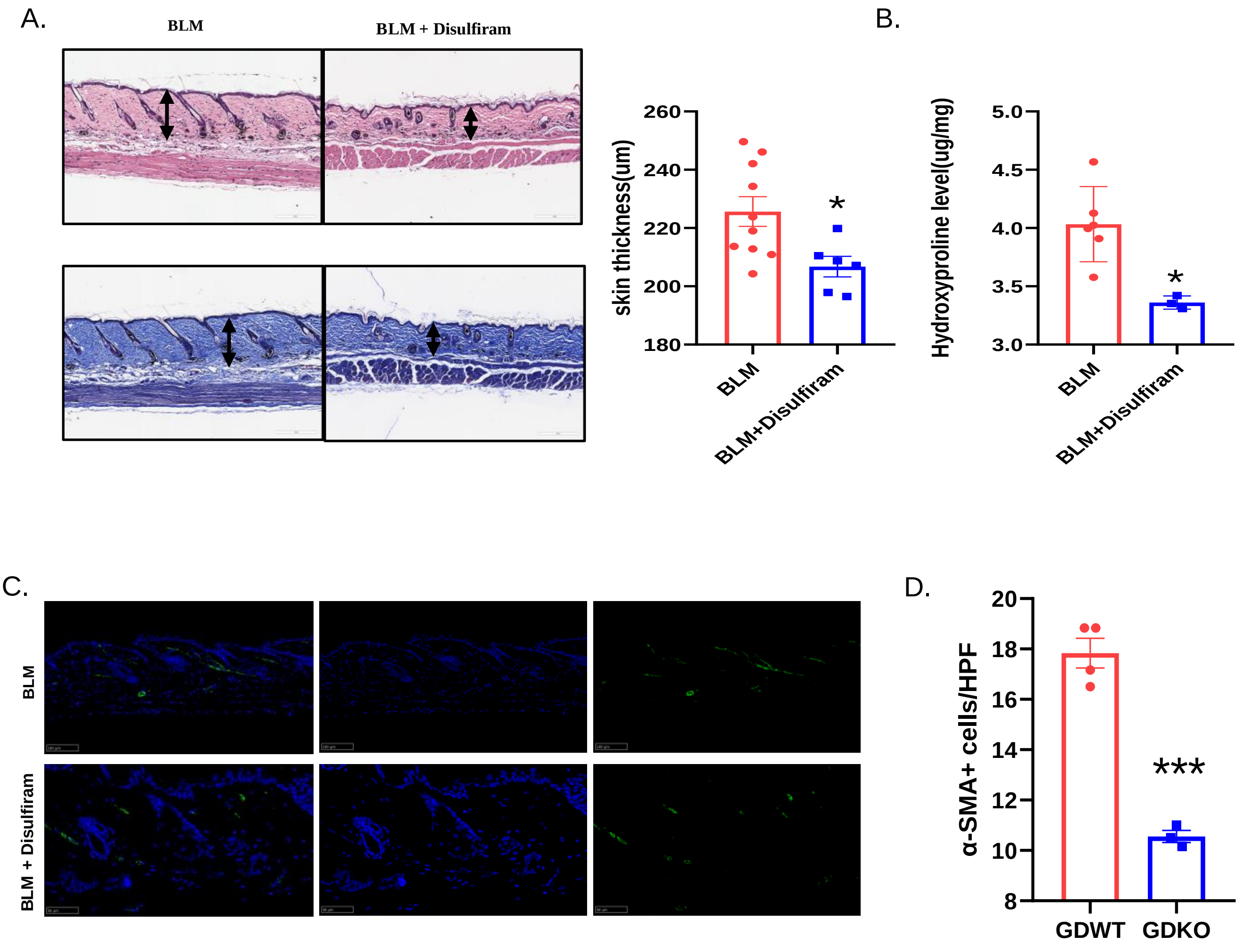

(A)Representative images with HE and Masson staining. Scale bar: 100μm, quantitation of dermal thickness (n=10, 6), and (B)hydroxyproline content demonstrating decreased dermal thickness in Disulfiram administration group (n=6, 3), *P<0.05, **p<0.01, ***P<0.001, *P<0.05(analyzed by t-test). (C) Myofibroblasts staining by anti-α-SMA antibody were counted in per high power field. (D)Statistical analysis of the number of α-SMA positive cells per high power field(n=4, 3), results are mean ± SEM, ***P < 0.001(analyzed by t-test).

Figure S3. Gating strategy of cytokine stimulation in T cells in flow cytometry.

A.

spleen

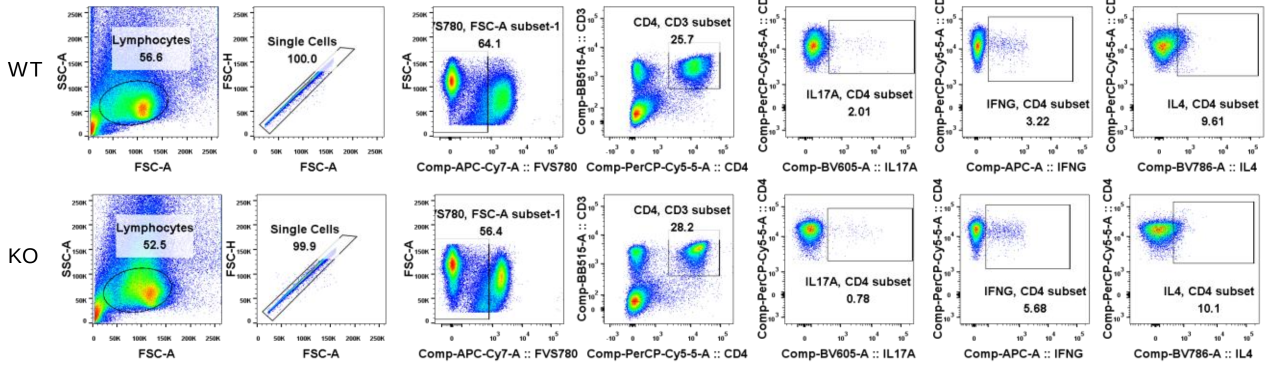

B.

Lymph nodes

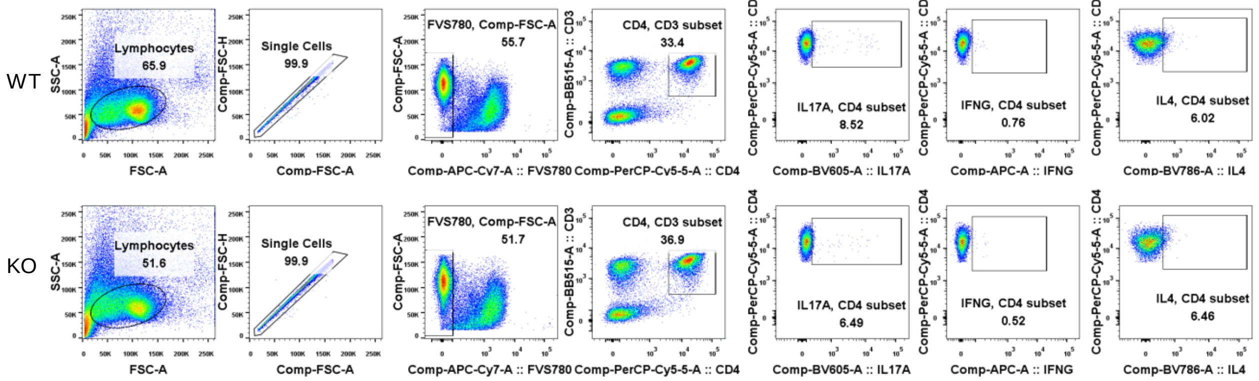

C.

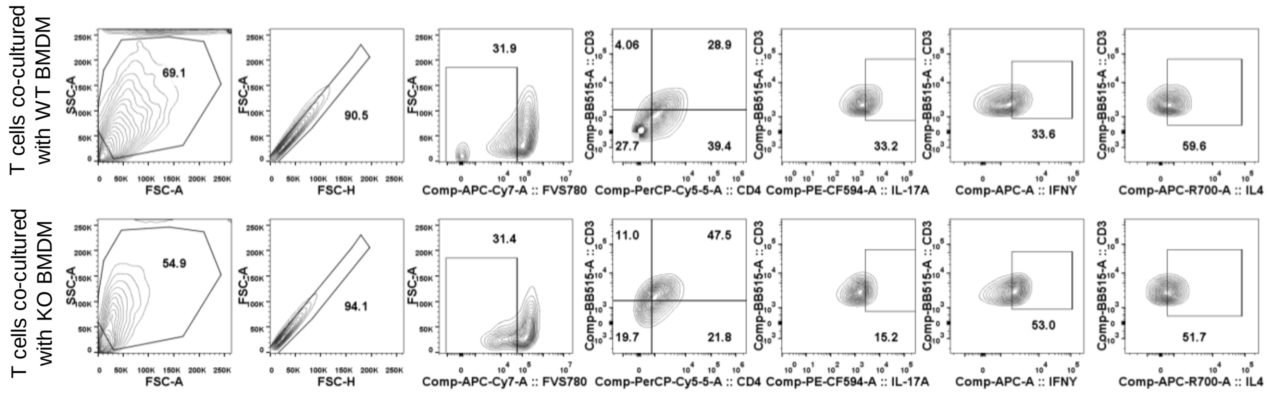

(A-C) Representative image of flow cytometry showing the gating strategy of cytokine stimulation in T cells.

Supplementary Table 1. Demographics and characteristics of included patients.

No.	Sex	Age(years)	Site of collection	subtype	Disease stage	Onset	Cleaved GSDMD
1.	F	4	Abdomen	LoS / Plaque	Early stage	6 months	+
2.	M	10	Waist	LoS / Plaque	Early stage	4 months	+
3.	F	30	Upper limb	LoS / Plaque	Early stage	3 months	+
4.	M	4	Back	LoS/ Plaque	Early stage	5 months	+
5.	M	27	scalp	LoS/ General	Early stage	6 months	+
6.	F	35	Neck	LoS/ Plaque	Early stage	2 years	+
7.	F	25	Upper limb	LoS/ Plaque	Early stage	1 years	+
8.	F	27	Lower limb	LoS/ Plaque	Early stage	3 years	+
9.	F	65	Buttock	SSc	Early stage	2 years	+
10.	M	46	Chest	SSc	Early stage	6 months	+
11.	F	48	Lower arm	SSc	Early stage	1 years	+
12.	F	44	Lower arm	SSc	Early stage	6 months	+
13.	M	36	Face	SSc	Non-early stage	5 years	-
14.	M	37	Back	LoS/ Plaque	Non-early stage	4 years	-
15.	F	15	Lower limb	LoS/ Linear	Non-early stage	3 years	-
16.	F	31	Chest	LoS/ Linear	Non-early stage	6 months	-
17.	F	5	Lower arm	LoS/ Linear	Non-early stage	7 months	-
18.	M	32	Face	LoS/ Plaque	Non-early stage	12 years	-
19.	F	57	Lower arm	SSc	Non-early stage	2 years	-

Supplementary Table 2. Demographics and characteristics of healthy control.

No.	Sex	Age(years)	Site of collection	Cleaved GSDMD
1	F	21	Buttock	-
2	M	37	Back	-
3	F	34	Back	-
4	F	51	Abdomen	-
5	F	28	Lower limb	-
6	M	31	Shoulder	-

Abbreviations: F, female; M, male.

Supplementary Table 3. Primers used in this study

Name	Sequence
mGAPDH-F	AGGTCGGTGTGAACGGATTTG
mGAPDH-R	TGTAGACCATGTAGTTGAGGTCA
mIL-18-F	GACTCTTGCGTCAACTTCAAGG
mIL-18-R	CAGGCTGTCTTTTGTCAACGA
mIL-1β-F	GCCACCTTTTGACAGTGATGAG
mIL-1β-R	GACAGCCCAGGTCAAAGGTT
mGSDMD-F	AGTGCTCCAGAACCAGAACC
mGSDMD-R	ACTCTGCCCTGAATGTTCCC
mTGF-β1-F	CTCCCGTGGCTTCTAGTGC
mTGF-β1-R	GCCTTAGTTTGGACAGGATCTG
